# Role of serum S100B and PET-CT in follow-up of patients with cutaneous melanoma

**DOI:** 10.1186/1471-2407-11-328

**Published:** 2011-08-02

**Authors:** Barbara Peric, Ivana Zagar, Srdjan Novakovic, Janez Zgajnar, Marko Hocevar

**Affiliations:** 1 Institute of Oncology, Ljubljana, Slovenia

## Abstract

**Background:**

Increased level of serum S100B can serve as a marker of metastatic spread in patients with cutaneous melanoma (CM). In patients with elevated S100 B and/or clinical signs of disease progression PET-CT scan is a valuable tool for discovering metastases and planning treatment.

The aims of this study were to determine whether regular measurements of serum S100B are a useful tool for discovering patients with CM metastases and to evaluate the diagnostic value of PET-CT during the follow-up.

**Methods:**

From September 2007 to February 2010, 115 CM patients included in regular follow up at the Institute of Oncology Ljubljana were appointed to PET-CT. There were 82 (71.3%) patients with clinical signs of disease progression and 33 (28.7%) asymptomatic patients with two subsequent elevated values of S100B. Sensitivity, specificity, positive and negative predictive value (PPV, NPV) of S100B and PET-CT were calculated using standard procedures.

**Results:**

Disease progression was confirmed in 81.7% of patients (in 86.5% of patients with clinical signs of disease progression and in 69.7% of asymptomatic patients with elevated S100B). Sensitivity, specificity, PPV and NPV of S100B was 33.8%, 90.9%, 96.0% and 17.5% in patients with clinical signs of disease progression. In 20.0% of patients increased serum S100B was the only sign of disease progression. Sensitivity and PPV of S100 in this group of patients were 100.0% and 69.7%.

With PET-CT disease progression was diagnosed in 84.2% of symptomatic patients and in 72.7% of asymptomatic patients with elevated S100B. The sensitivity, specificity, PPV and NPV of PET-CT for symptomatic patients was 98.5%, 90.9%, 98.5% and 90.9% and 100%, 90.0%, 95.8% and 100% for asymptomatic patients with elevated S100.

**Conclusions:**

Measurements of serum S100B during regular follow-up of patients with CM are a useful tool for discovering disease progression in asymptomatic patients. The value of its use increases if measurements are followed by extended whole body PET-CT.

## Background

The prognosis of patients with cutaneous melanoma (CM) is determined by histology of the primary tumour and by the presence and extent of metastatic disease. Available laboratory, radiographic and nuclear medicine techniques are important components of patient evaluation but their usefulness is being constantly questioned by clinicians.

During the last decade many CM associated blood compounds have been examined including melanoma associated antigens, cytokines, metalloproteinase, angiogenesis factor and others. Of all these compounds, S100 is at the moment the only widely applied CM biomarker [1]. More than 20 years ago, it was first demonstrated that melanoma cells can secrete a soluble form of S100 protein [2]. The term S100 refers to members of a multigene family of low-molecular-weight (9-13 kDa) calcium -modulated proteins [1]. The first one was originally isolated from the bovine brain tissue and named after its solubility in 100% saturated ammonium sulphate solution at neutral pH [1,3]. Currently there are 20 known members of structurally related and interacting proteins [4]. Two subunits S100 A and S100 B have the ability to form homodimers, heterodimers and oligodimers and are widely distributed in the peripheral and central nervous system but can be also expressed by cells such are chondrocytes, adipocytes and melanocytes. S100 proteins are involved in a large number of cellular activities such as signal transduction path, cell differentiation, motility, transcription and cell cycle progression [1].

S100B, formed by two B or A and B subunits, is currently best-studied CM biomarker. Increased serum levels of S100B are associated with presence of metastases and are described to correlate with reduced survival. They reflect tumour load, stage and prognosis and are believed to be an independent poor prognostic factor in CM [3]. Findings published by Mocellin even proved, that this serum marker influences patients survival independently of the TNM staging system [5]. S100B has also been used as a valuable marker in assessing patient's response to treatment [3]. Unfortunately abnormal circulating levels can also be found in liver and renal injury, and in various inflammatory conditions and infectious disease [1].

PET-CT is increasingly used for staging of melanoma patients. With its combination of metabolic and morphologic information, PET-CT has high accuracy for metastases and is superior to CT or MRI alone [6]. Sensitivity is highest (≥90%) for metastases that are > 1 cm in diameter, but tumour deposits as small as 0.6 cm can be seen in areas of low background activity. It is believed that PET-CT is most useful for identifying all metastatic sites of melanoma before deciding on operation of an apparently isolated lesion [7].

In Slovenia, a country with a population of two million, the prevalence of CM was 1302 male and 1920 female patients in December 2009. All patients with stage III and IV CM and a proportion of patients with stage I and II melanoma are engaged in regular follow-up at the Institute of Oncology Ljubljana, the only comprehensive cancer centre in the country.

The aims of this study were to determine whether regular measurements of serum S100B are a useful tool for discovering patients with CM metastases and to evaluate the diagnostic value of subsequent PET-CT during the follow-up.

## Methods

### Patients

From September 2007 to February 2010, 115 patients (45 female and 70 male) with CM were directed to extended whole body PET-CT, based on clinical signs of recurrent disease, increased serum S100B or both. All patients were engaged in a regular follow-up at the Institute of Oncology Ljubljana after being diagnosed with CM. Clinical stage of the patients at the time of diagnosis of CM was classified according to American Joint Committee on Cancer (AJCC) criteria. Most patients were diagnosed with localized stage I and II of CM (21 or 18.3% and 47 or 40.9% respectively), but 40 (34.8%) of patients were stage III and 5 (4.3%) were stage IV at the time of diagnosis. For two patients (1.7%) stage of the disease at the time of diagnosis could not be assessed reliably.

Initially 33 (28.7%) patients were treated only by wide excision, 39 (33.9%) patients with wide excision and sentinel node biopsy, while in 40 cases (34.8%) limphadenectomy was performed. There was a case of locally advanced anal melanoma treated with abdominoperineal resection, a case of isolated limb perfusion and a patient refusing all treatment after initial excision. Three patients diagnosed with stage IV disease received dacarbazine and two patients received only symptomatic treatment. All patients were followed at regular intervals, i.e. every 3 months in the first and second year after the diagnosis, every 6 months in the third to fifth year, and once a year after first 5 years. Patients were monitored at each visit for relapse by history, physical examination and laboratory tests including S100B. Disease progression was suspected based on clinical signs in 82 (71.3%) patients and based on increased S100B in 33 (28.7%) asymptomatic patients. There were 25 (21.7%) patients with both, increased S100B and clinical signs of advancing disease. In majority of patients (34.8%, 40/115) skin metastases were the first sign of progression. Twenty-four patients (20.9%) had palpable lymph nodes, 2.6% complained of persistent cough (3/115), 9.6% (11/115) of pain and in two cases (1.7%) mild gastro intestinal bleeding was present. In two patients, accidental radiological findings could not be ruled out as CM metastases by treating physicians. Mean time at which PET-CT followed measurement of serum S100B was 21.19 days.

All patients were informed about the research protocol and signed a consent form. The study was reviewed and approved by institutional Ethics Committee.

### S100B

Serum concentrations of S100B (heterodimers S100 A1B and S100 BB) were determined by the use of the electrochemiluminescence immunoassay (ECLIA). The immunoassays were analyzed on the Modular Analytics E170 immunoassay analyzer (Roche Diagnostics). Reference values were obtained from apparently healthy volunteers: 0.105 μg/L was determined as the upper normal value (Roche Diagnostics). In cases of increased tumour marker values, sampling and determination of the serum S100B were repeated for confirmation after two weeks. Only patients with elevated repeated values were appointed to subsequent PET-CT scan.

### PET-CT scan

Extended whole body PET-CT scan was performed in cases where distant metastases were suspected due to elevated serum S100B or due to clinical signs of the disease. The scans were performed after a six-hour fast and when the patients were prepared according to recommendations (EANM guidelines) [8]. A hybrid PET/CT camera Philips Gemini GXL was used and FDG was administrated in dosage of 5.18 MBq (0.14 mCi)/kg body weight. The interval between FDG administration and scanning was 60 minutes ± 10 minutes. Low-dose CT images were obtained (150 mAs, 3 mm slices) without oral or intravenous contrast. Acquired images were viewed using a Brilliance and Medic View 3D display. PET was fused to low-dose CT after correction for attenuation. The PET-CT scans were reviewed by an experienced nuclear medicine physician. The presence of abnormal FDG accumulation in the images in combination with their size and intensity was evaluated. Under normal circumstances a standard uptake value (SUV) of 2.5 was considered normal.

### Statistics

The McNemar symmetry chi square test was used to compare performance of the S100B with that of PET-CT. Sensitivity, specificity, positive predictive value (PPV) and negative predictive value (NPV) of S100B and PET-CT were calculated using standard procedures. Separate analyses were made for patients with no clinical signs of the disease and for patients with CM stage I and II at the time of diagnosis. All statistical analyses were performed using the Statistical Package for Social Sciences (SPSS) for Windows version 15.0.

## Results

115 patients suspected of disease progression based on clinical signs (82, 71.3%) and/or increased levels of serum S100B (33, 28.7%) were appointed to extended whole body PET-CT at the Institute of Oncology Ljubljana from 2007 until the beginning of 2010. The mean age of the patients at the time PET-CT was performed was 60.8 (ranged from 16.3 to 86.8) years. The group comprised of 39.1% female and of 60.9% male patients.

Out of 115 patients, disease progression was diagnosed in 94 (81.7%) patients. Metastases were confirmed by fine needle aspiration cytology in 34 (36.2%), with CT or MRI in 11 cases (11.7%), with ultrasound or radiological finding in 11 cases (11.7%) and based on the histology report in 38 cases (40.4%) prior to implementation of appropriate treatment.

As far as the rest of the patients are concerned, there were 3 patients with palpable lymph nodes, 6 patients with pain and 2 patients with suspicious radiological findings, however the metastases could not be confirmed. Altogether 21 patients proved to be disease free at that time (18.3%).

The majority of patients with skin metastases (distant and in transit included) had normal level of S100B. Patients with lymph node metastases had increased S100B in 50.0% of cases compared to 57.1% of patients with distant visceral metastases (Table 1). Table 1 shows location of discovered metastases and results of S100B measurements for all 115 patients.

**Table 1:** Confirmed location of CM metastases and value of serum S100B

*Metastases*				
**Increased serum S100B**	**Skin** **(%)**	**Regional lymph nodes (%)**	**Distant visceral** **(%)**	**Absent** **(%)**

**Yes**	8 (34.8)	11 (50.0)	28 (57.1)	11 (52.4)

**No**	15 (65.2)	11 (50.0)	21 (42.9)	10 (47.6)

**Total**	23	22	49	21

Out of 82 patients with suspicious clinical signs, metastases were confirmed in 71 (86.5%). The majority of these patients did not have increased values of serum S100B (47/71, 66.2%). In eleven patients presented with clinical signs, metastases were not discovered.

Symptomatic patients had elevated S100B levels in 30.5% of cases (25/82). The mean value of S100B marker in that group of patients was 0.603 μg/L (median value 0.228 μg/L). For symptomatic patients without increased S100B, the mean value of serum marker was 0.051 μg/L (median value 0.046 μg/L). In 33 patients without clinical signs of disease progression, the mean value of S100B was 0.652 μg/L (median value 0.255 μg/L).

In 33 patients with no symptoms but with an increased S100B, metastatic disease was confirmed in twenty three patients (69.7%) and 10 patients were healthy. Based solely on increased S100B, metastases were discovered in 20.0% (23/115) of all cases.

The PET-CT pointed out suspicious lesions in 95/115 (82.6%) patients. In 20 (17.4%) patients PET-CT was negative. False positive results with SUV 15.2 and 8.0 were obtained in two patients. Metastases were actually confirmed in 94 (81.7%) out of 115 patients. The χ^2 ^value for McNemar test comparing S100B and PET-CT was 21.9 with p < 0.0001 respectively.

Patients with normal S100B level but suspicious clinical signs, had metastases diagnosed with PET-CT in 84.2% of cases (48/57). Disease progression was later actually confirmed in 47 of those patients. In 9 cases PET-CT was negative and patients were proved to be disease free.

If patients had increased S100B level (58 patients, 25 with symptoms and 33 without), PET-CT diagnosed suspicious lesions in 47 (81.0%) cases. In 11 cases with increased S100B, PET-CT was negative. In only one of those cases metastases were present later on follow-up. Out of 33 asymptomatic patients with increased S100B suspicious lesions were diagnosed in 72.7% of cases.

Based on those results 98.9% sensitivity, 90.4% specificity, 97.8% PPV and 95.0% NPV of PET-CT was calculated for the whole group of patients (Table 2).

**Table 2:** Sensitivity, specificity, PPV and NPV of serum S100B and PET-CT

*All pts* *(115)*				
	**PPV %** **(N)**	**NPV %** **(N)**	**Sensitivity % (N)**	**Specificity % (N)**

**S100B**	81.0 (47/58)	17.5 (10/57)	50.0 (47/94)	47.6 (10/21)

**PET-CT**	97.8 (93/95)	95.0 (19/20)	98.9 (93/94)	90.4 (19/21)

***Pts with clin. sign*** ***(82)***				

	**PPV %** **(N)**	**NPV %** **(N)**	**Sensitivity % (N)**	**Specificity % (N)**

**S100B**	96.0 (24/25)	17.5 (10/57)	33.8 (24/71)	90.9 (10/11)

**PET-CT**	98.5 (70/71)	90.9 (10/11)	98.5 (70/71)	90.9 (10/11)

***Asymptomatic pts*** ***(33)***				

	**PPV %** **(N)**	**NPV %** **(N)**	**Sensitivity % (N)**	**Specificity % (N)**

**S100B**	69.7 (23/33)	0 bias	100.0 (23/23)	0 bias

**PET-CT**	95.8 (23/24)	100.0 (9/9)	100.0 (23/23)	90.0 (9/10)

Analysis was repeated for 68 patients with localized disease (stage I or II) at the time of the first visit. In 54/68 patients disease progression was confirmed during the follow-up. Among 48 patients with clinical signs of disease progression, there were 15 (31.2%) individuals where S100B was also increased. In majority of patients with clinical signs, metastases were confirmed (40/48, 83.3%).

Table 3 presents specificity, sensitivity and predictive values for patients with localized disease.

**Table 3:** Sensitivity, specificity, PPV and NPV of serum S100B and PET-CT for patients with stage I or II

*Pts with stage I or II* *(68)*				
	**PPV %** **(N)**	**NPV%** **(N)**	**Sensitivity% (N)**	**Specificity% (N)**

**S100B**	80.0% (28/35)	21.2% (7/33)	51.8% (28/54)	50.0% (7/14)

**PET-CT**	98.2% (54/55)	100.0% (13/13)	100.0% (54/54)	92.8% (13/14)

***With clin. sign*** ***(48)***				

	**PPV%** **(N)**	**NPV%** **(N)**	**Sensitivity% (N)**	**Specificity% (N)**

**S100B**	93.3 (14/15)	21.3 (7/33)	65.0 (26/40)	87.5 (7/8)

**PET-CT**	97.5 (40/41)	100.0 (7/7)	100.0 (40/40)	87.5 (7/8)

***Asymptomatic pts*** ***(20)***				

	**PPV%** **(N)**	**NPV%** **(N)**	**Sensitivity% (N)**	**Specificity% (N)**

**S100B**	70.0 (14/20)	0 bias	100.0 (14/14)	0 bias

**PET-CT**	100.0 (14/14)	100.0 (6/6)	100.0 (14/14)	100.0 (6/6)

## Discussion

Since the prognosis of patients with CM is determined by the extent of metastatic disease, serum biomarker and imaging studies using radiographic and nuclear medicine techniques are an important component of patient evaluation [7].

Almost 10 years ago researchers suggested that measuring S100B after primary local surgical therapy may allow early recognition of disease progression. It was also suggested that an increasing S100B, followed by a positive emission tomography (PET) scan and appropriate treatment, could lead to an increased life expectancy of patients with CM [9]. Despite being shown, that S100B levels correlate with disease staging, the extent of metastatic spread and disease progression, the value of it's measuring in follow-up is still controversial. This is true especially for early stages of the disease. The diagnostic sensitivity of serum S100B in patients with stage I and II CM has been reported to be 15% or less compared to 60-85% sensitivity for stage IV CM. For patients with regional metastases, sensitivity varies from 10% to 50% [1,7].

We analyzed a group of 115 patients appointed to PET-CT due to increased serum S100B value (33 pts) or clinical signs of disease progression encountered during regular follow-up (82 pts). In our study patients with stage I or II of CM had 51.8% sensitivity and 50.0% specificity of S100B. PPV of S100B for this group of patients was 80.0% and NPV was only 21.2%. A similar study conducted in 2002 showed sensitivity between 3% and 9% for stage I and II. The study described a PPV of 17% for stage I and 37% for stage II and specificity was 92%. Based on that, the authors concluded that S100B could not be a reliable marker of disease dissemination at early stages [10].

The sensitivity of increased serum S100B for the whole group of patients (115 pts) regardless of the stage of the disease in our study was 50.0%, specificity 47.6%, PPV 81.0% and NPV 17.5%. In studies which included patients with clinical symptoms being either present or absent the sensitivity was 38% with PPV 93% and 37% respectively [10,11]. Our results for the whole group of patients did not differ much from the results for the group of patients with stage I or II, which could indicate that successful use of marker is not conditioned by the stage of the disease. The sensitivity and specificity of the test in British study was 74% and 87% with PPV of 87% and NPV of 76% for the group of patients regardless of the stage of CM. They suggested that S100B may accurately reflect the activity of the disease at the sampling time. The surprising finding of that study was, that the conclusion is not true in the presence of cerebral metastases even if additional organs were involved [12].

It has been observed by other authors that location of the metastases influences the level of S100B. Authors report that between 41 to 68% of patients with stage III disease do not have an increased serum marker [11,13-15]. In our study 50.0% of patients with metastases limited to the lymph nodes and 42.9% of patients with distant metastases did not have the increased level of serum marker. The result could be a consequence of a smaller tumour burden in the case of stage III disease as it has been proposed by others [13,15]. Another possibility would be that primary tumours have different expression patterns of protein S100B. In the case of the metastatic disease, serum levels would depend on the ability of CM cells to produce a serum marker, which would explain false negative results in the case of distant metastases [12,13].

Comparing increased levels of serum S100B from different studies has one major obstacle, namely researchers are using different immunoassays with slightly different cut off values of serum S100B. Different assays used in clinical practice were compared and it was determined that the correlation among assays is satisfactory with correlation index of 0.9 or higher for most assays. Nevertheless, data from the assays using bovine brain solution and those standardized against human recombinant S100B can not be superimposed. Difficulties are also encountered comparing upper cut off values ranging from 0.09 to 0.13 μg/l, which indicates the need for international agreement regarding the use of uniform reference standards for successful comparison of different studies [16].

All patients in our study were appointed to extended whole body PET-CT. Eleven patients (11/115, 9.5%) had increased level of S100B, but no metastases were proven on PET-CT or by clinical follow up. The number of reported false positive results of S100B in other studies ranges from 17 to 41% of cases [15]. There are few possible explanations for false positive results. Elevated levels of S100B had been observed after head trauma and stroke and can reflect a brain injury or dysfunction of blood-brain barrier. Significantly higher serum levels can be found in patients with liver cirrhosis or renal failure [15]. Interestingly 25% of healthy individuals can also have an increased S100B level, the fact that could be explained by an increased serum S100B level measured after UVB exposure of some individuals [17].

The main limitation of our study is that patients were appointed to PET-CT based on the increased S100B or clinical suspicion of disease progression which represents a selection bias influencing NPV and specificity of S100B. Published retrospective studies also describe groups of patients inspected with PET-CT due to increased S100B and report similar bias [15]. Our group of patients was chosen because we were interested in clinical value of S100B measurements in conjunction with PET-CT during regular follow up. A study published by Aukema concentrated on patients with normal findings at physical examination and increased serum S100B level. At the cut off value similar to one used by us, PPV of the S100B was reported to be 50%. The PPV observed by us was 69.7%. The sensitivity and PPV of both studies prove a serum measurement of S100B as a helpful but not perfect method for follow up of all CM patients [18].

The sensitivity of PET-CT in our study was 98.9% with 90.4% specificity for the whole group of patients and 100% with 90.0% specificity if clinical signs were absent. The accompanying PPV was 97.8% in the first group and 95.8% in the subgroup of patients. Aukema reported a sensitivity of 100%, specificity of 83%, PPV of 85% and NPV of 100% [18]. The role of PET-CT in the management of patients with CM evolved rapidly. It has been shown, that PET-CT enables more accurate staging of distant metastases than PET or CT alone (98% compared to 93% and 84% respectively), and is more accurate in diagnosing lymph node metastases (98% compared to 86%) [7,15]. Diagnosing metastases with PET-CT is overall more accurate then CT or MRI alone and at the same time provides valuable information to the surgeon planning an operative procedure [7,18]. Out of 95 patients being diagnosed with suspicious lesions on PET-CT, two proved to be a false positive (2.1%). Other authors also report false positive results [15,18]. In our study one false positive result was a case of lesion with diameter of 1 cm suspected to be a metastasis. It has been stated in a recent report describing staging of CM that sensitivity of PET-CT is highest for metastases that are > 1 cm in diameter and it is recognized that in the case of melanoma, the sensitivity of the method declines when the diameter of tumour is less then 6 mm [7,8]. In the second false positive PET-CT result, careful examination of the patient's record revealed a chronic wound at the location of the increased FDG uptake. It is known that increased uptake is observed not only in neoplastic lesions but also in granulation tissue, infection and other inflammatory processes. In order to use PET-CT successfully it is necessary to evaluate the size and intensity of abnormal FDG focal accumulation with patients history and physical examination [8].

Due to short follow-up, we did not investigate how the outcome of regular measurements of serum S100B and subsequent PET-CT influences overall survival of our patients. It is known that increased S100B levels are related to prognosis and survival of patients with CM. A meta-analysis of twenty-two articles published until 2008 proved S100B as an independent prognostic factor of survival with special regard to stage I-III patients [5]. In advanced disease elevated levels of S100B also demonstrate a strong correlation with reduced survival as patients with stage III or IV disease and low S100B have shown statistically significant survival advantage compared to patients with elevated tumour marker [1,15]. Whether PET-CT can act as an independent prognostic factor remains to be elucidated [15].

We confirmed metastases in 20.0% of patients based on the increased serum S100B as the only preceding sign of disease progression (23/115). Since the analysis of serum S100B showed the highest sensitivity and specificity of all tumour markers examined in CM and it is fairly inexpensive, numerous clinicians include its measurement in routine follow up of CM patients [7,19]. In the case of disease progression monitoring, rising level of serum S100B is believed to be a specific and sensitive marker of disease relapse that can detect metastases 5-23 weeks before other clinical and radiological methods. During routine follow-up, elevated S100B levels were noted 6 months before ultrasound and chest X-ray were able to detect disseminated disease [1]. Serum S100B can be used as a marker of disease progression which together with subsequent PET-CT enables clinicians early diagnosis of metastases during regular follow-up.

If there is an actual benefit of carefully planned follow-up schedules for CM patients remains to be elucidated. A study conducted by German researchers proved a gain in survival time for the detection of metastasis at an early phase of development beyond the effect of lead time bias. Namely the 10-year overall survival probability was increased significantly for patients with early phase metastases [20].

## Conclusion

Measurements of serum S100B during regular follow-up of patients with CM are a useful tool for discovering disease progression in asymptomatic patients. This applies equally to patients with localized disease (stage I and II) as to patients with stage III or IV. The value of its use increases if measurements are followed by extended whole body PET-CT. If the approach described in our study has any influence on patients' survival will be validated in the future.

## Competing interests

The authors declare that they have no competing interests.

## Authors' contributions

BP participated in patient recruitment and data set up, performed the statistical analysis and drafted the manuscript. IZ interpreted the PET-CT scans and helped to draft the manuscript. SN participated in the design of the study and helped to draft the manuscript. JZ participated in patient recruitment, study design and helped to draft the manuscript. MH conceived of the study, participated in its design, coordination and patient recruitment and drafted the manuscript. All authors read and approved the final manuscript.

## Pre-publication history

The pre-publication history for this paper can be accessed here:

http://www.biomedcentral.com/1471-2407/11/328/prepub
